# An Additional Criterion to Appraisal of Guidelines for Research and Evaluation II Might Increase the Efficiency in Guideline Development for Rare Diseases: Results of a National Delphi Study

**DOI:** 10.1002/gin2.70040

**Published:** 2025-09-07

**Authors:** Iméze Jorieke Hieltjes, Charlotte M. W. Gaasterland, Roel Bakx, Silvia C. W. van Breukelen, Robbert J. H. Ensink, Charlotte van Esch, Raoul C. M. Hennekam, Ilse van Herk, Marieke Hermsen, Anja M. C. Horemans, Hester Rippen, Bart P. C. van de Warrenburg, Anne‐Marie van Wermeskerken, Johanna H. van der Lee

**Affiliations:** ^1^ Knowledge Institute of the Dutch Association of Medical Specialists Utrecht the Netherlands; ^2^ Amsterdam UMC, Emma Children's Hospital University of Amsterdam Amsterdam the Netherlands; ^3^ Department of Epidemiology Leiden University Medical Center Leiden the Netherlands; ^4^ Department of Pediatric Surgery Amsterdam UMC, Emma Children's Hospital Amsterdam the Netherlands; ^5^ Guidelines Advisory Committee of the Dutch Association of Medical Specialists Utrecht the Netherlands; ^6^ VSOP Utrecht the Netherlands; ^7^ Gelre Ziekenhuizen Apeldoorn the Netherlands; ^8^ Spierziekten Nederland Baarn the Netherlands; ^9^ University of Amsterdam Amsterdam the Netherlands; ^10^ Dutch Pediatric Association Utrecht the Netherlands; ^11^ Dutch Association of Medical Specialists Utrecht the Netherlands; ^12^ Kind en Ziekenhuis Utrecht the Netherlands; ^13^ Department of Neurology Radboud University Medical Center Nijmegen the Netherlands; ^14^ Flevoziekenhuis Almere the Netherlands

**Keywords:** clinical practice guidelines, Delphi consensus, guidance documents, healthcare quality, quality documents, rare diseases

## Abstract

**Introduction:**

Rare diseases significantly impact patients, and there is a need for clinical practice guidelines (CPGs) or other guidance documents as well as tools to assess their trustworthiness.

**Objective:**

The goal was to define a minimum set of criteria for the appraisal of guidance documents for rare diseases. Guidance documents include CPGs, but also other, less stringently developed documents to guide clinical practice.

**Study Design:**

In 2022, we started a Delphi consensus procedure to develop criteria specifically for rare disease guidance documents. Participants were 23 delegates from 18 medical specialists' associations, 3 patient organizations for rare diseases, and 2 other medical organizations in the Netherlands. The Delphi procedure consisted of three rounds. In the first round, the participants were asked whether six prespecified criteria, selected from AGREE II, were relevant, and to add other important criteria. In the second round, all collected criteria were prioritized, and in the third round, the criteria with the highest priority were ordered by the participants. In an online meeting, the remaining criteria were reformulated.

**Results:**

Six of the seven final criteria correspond to the AGREE II criteria. The participants proposed a new criterion focusing on efficient use of limited resources, namely the inventory of problem areas as a basis for specific clinical questions to which the recommendations would provide answers.

**Conclusion:**

The current set of criteria for rare disease guidance documents helps evaluate the trustworthiness and enhance equitable healthcare quality for rare disease patients. Although the aim was that the minimum set of criteria reduces the time and effort for developing quality criteria for rare diseases, the participants' perceived need for methodological rigour prevailed.

## Summary

- Dutch stakeholders of guidelines for rare diseases, including patient representatives and medical specialists, have suggested a new criterion to enhance the efficient use of limited resources for developing guidance documents for rare diseases.
- Guidance documents include, but are not limited to, clinical practice guidelines.
- They propose creating a list of problem areas to help focus on specific clinical questions.
- In addition to this new criterion, there are six other criteria that were agreed upon through a Delphi consensus process.
- These six criteria correspond to the existing AGREE II criteria, which are used to evaluate the quality of guidelines.
- Together, these seven criteria help users determine if the guidelines for rare diseases are trustworthy, even if they do not meet the Dutch definition of guidelines.
- The involvement of diverse stakeholders, including patient representatives, ensures that these criteria are practical and relevant in real‐world situations.

## 1 Introduction

A rare disease affects only a small percentage of the population but exerts a significant impact on the lives of affected individuals. In Europe, a disease is classified as rare if it affects no more than 1 person per 2000 [1, 2, 3]. Currently, there are approximately six to seven thousand known rare diseases, with new diseases being discovered frequently [4]. Hence, while the prevalence of individual rare diseases is low, the cumulative impact of rare diseases on the overall population is substantial [1]. In the European Union (EU), more than 30 million people suffer from a rare disease [4]. However, despite this collective impact, rare diseases often receive less attention and research focus compared to more common diseases, primarily due to the complexities associated with studying rare diseases [5]. This may lead to substandard quality of care for patients with rare diseases, even though the European Commission has stated that quality of care should be similar for every patient, regardless of the prevalence of the disease [6].

To improve the quality of care for patients, particularly for rare diseases, clinical practice guidelines (CPGs) play an important role [7]. These documents support clinical decision‐making and contribute to improved care, increased transparency and reduced unwarranted practice variation [8]. CPGs provide recommendations for optimal care based on the best available evidence [9]. The Dutch Association of Medical Specialists (in Dutch Federatie Medisch Specialisten) defines a CPG as a document with recommendations, to support healthcare professionals and healthcare users, aimed at improving the quality of care, based on systematic summaries of scientific research and considerations of the advantages and disadvantages of the various healthcare options, supplemented with expertise and experiences of healthcare professionals and healthcare users [8]. A variety of terms are used to denote tools for knowledge translation and supporting (shared) decision‐making, with CPGs being one among them [10].

The Dutch Association of Medical Specialists uses the GRADE methodology [11] and its guidelines comply with the Appraisal of Guidelines for Research and Evaluation II (AGREE II) criteria [12].

The AGREE II instrument [12] is a checklist of 23 items in 6 domains designed to assess the methodological quality and transparency of CPGs.

Not all documents described as guidelines meet the criteria for CPGs of the Dutch Association of Medical Specialists. The methodological quality of these guidelines varies considerably, in particular for rare diseases [13]. Even though methods have been developed for the development and maintenance of guidelines for rare diseases that align with the internationally accepted GRADE methodology, these methods are even more labour‐intensive than the methods for common diseases, making it even harder to develop CPGs for rare diseases with limited resources [7]. Given the limited resources, it will never be possible to develop a CPG that complies with all 23 items of the AGREE II checklist for each rare disease. However, there is still a need for guidance documents to support decision‐making and clinical care for patients with rare diseases. In this article, we will use the term ‘guidance documents’ as an umbrella term for knowledge documents and documents aimed at improving the quality of care, with CPGs being a subset.

The goal of this study was to reach consensus on a minimum set of criteria, specifically setting criteria for evaluating guidance documents for rare diseases, which may leave room for more efficiently developed guidance documents for rare diseases while ensuring methodological rigour.

## 2 Methods

This project was initiated by the Dutch Pediatric Association, funded by the Dutch Medical Specialists Quality Fund (Stichting Kwaliteitsgelden Medisch Specialisten, SKMS), and supported by the Knowledge Institute of the Dutch Association of Medical Specialists. All stakeholders in the field of rare diseases and CPG development in the Netherlands were invited to send delegates from their organizations. A Delphi consensus procedure was organized to reach consensus and prioritize a minimum set of criteria for guidance documents for rare diseases for the specialists' associations to decide about endorsement of the guidance documents and publication on their official websites [14]. In the first round, the participants were asked whether six prespecified criteria were relevant, and to add other important criteria. In the second round, all collected criteria were prioritized, and in the third round, the remaining criteria with the highest priority were ordered by the participants. Subsequently, in an online meeting, the remaining criteria were reformulated until full consensus was reached. The scientific associations and organizations mentioned in Table 1 sent delegates with expertise in their specific fields.

**Table 1:** Organizations represented in the panel in the Delphi procedure.

Organization (in Dutch)	English translation
Nederlandse Vereniging voor Kindergeneeskunde (NVK)	Dutch Pediatric Association
Vereniging Klinische Genetica Nederland (VKGN)	Association of Clinical Genetics Netherland
Nederlandse Vereniging voor Obstetrie en Gynaecologie (NVOG)	Dutch Society for Obstetrics and Gynaecology
Nederlandse Internisten Vereniging (NIV)	Dutch Society of Internal Medicine
Nederlandse Vereniging voor Neurologie	Netherlands Society of Neurology
Patiëntenkoepel voor zeldzame en genetische aandoeningen (VSOP)	Patient Umbrella Organization for Rare and Genetic Disorders (Netherlands)
Stichting kind en ziekenhuis (K&Z)	Child and Hospital Foundation
Spierziekten Nederland (SN)	Dutch Society for Neuromuscular Diseases
Richtlijnen advies commissie	Guidelines Advisory Board of the Dutch Association of Medical Specialists
Verpleegkundigen & Verzorgenden Nederland (V&VN)	Netherlands Nurses & Nursing Assistants Association
Vereniging Innovatieve Geneesmiddelen (VIG)	Association of Innovative Medicines
Nederlands Oogheelkundig Gezelschap (NOG)	Dutch Opthalmological Society
Nederlandse Vereniging voor Anesthesiologie (NVA)	Dutch Association of Anesthesiology
Nederlandse Vereniging van Artsen voor Longziekten en Tuberculose (NVALT)	Dutch Association of Physicians for Pulmonary Diseases and Tuberculosis
Nederlandse Vereniging voor Dermatologie en Venereologie (NVDV)	Dutch Society of Dermatology and Venereology
Nederlandse Vereniging voor Klinische Chemie en Laboratoriumgeneeskunde (NVKC)	Netherlands Society for Clinical Chemistry and Laboratory Medicine
Nederlandse Vereniging voor Plastische Chirurgie (NVPC)	Netherlands Society of Plastic Surgery
Nederlandse Vereniging voor Reumatologie (NVR)	Dutch Society for Rheumatology
Nederlandse Vereniging voor Cardiologie (NVVC)	The Netherlands Society of Cardiology
Nederlandse Vereniging voor Heelkunde (NVvH)	Dutch Society for Surgery
Nederlandse Vereniging van Ziekenhuis Apothekers (NVZA)	Dutch Association of Hospital Pharmacists
Nederlandse Vereniging van Revalidatieartsen (VRA)	Netherlands Society of Rehabilitation Medicine

The panel consisted of 23 delegates from medical as well as patient organizations. An overview of the organizations represented in the panel of the Delphi procedure is shown in Table 1.

In the first round, the organizing group proposed the set of minimum criteria shown in Table 2, loosely based on the quality criteria of the Dutch Guidelines Database [8], which correspond with a selection of criteria from AGREE II.

**Table 2:** Criteria proposed in the first round with corresponding AGREE II items, random order.

Proposed criteria	Corresponding AGREE II items
1. Transparency of authors and methods	10, 12
2. Involvement of all relevant parties	4
3. Use of a code for the prevention of improper influence due to conflicts of interest	22, 23
4. Systematic search and review of literature	7, 8
5. Authorization by relevant scientific societies	13
6. Approval by a relevant patient organization	5

These criteria were sent to all panel members in expectation of a binary answer for every criterion, in terms of its necessity for evaluating a guidance document for rare diseases.

The panel members were also asked to add criteria that they considered essential to evaluate guidance documents for rare diseases. The response time for the questionnaire was 3 weeks.

The second round consisted of the evaluation of the criteria derived from the first round. All criteria collected during the first round were presented to the panel members. They were asked to assess the importance of each criterion for a guidance document for rare diseases using the numbers 1–10, with a higher number indicating greater importance. The average and range of the scores of all respondents were calculated for each criterion. To determine the agreement between experts for each criterion, the formula (10 − range) was used. A higher value of (10 − range) corresponds to a smaller range and more agreement. A combination score for each criterion was computed by adding up the average score and (10 − range). The maximum score possible was 20 and the minimum score was 1. Criteria with a combination score ≥ 10, as an arbitrary limit, were included in the third round.

In the third round, the selected criteria were categorized according to the domains of AGREE II, and criteria that were considered duplicates were combined. The panel members were asked to rank the criteria in order of importance, with the understanding that the exact wording of the criteria could be revised later. In the subsequent consensus meeting, the ranks per criterion were visualized in histograms to show the level of (dis)agreement. During the consensus meeting the number of criteria was reduced and the exact wording was decided by discussion until all panel members agreed, voting was not necessary.

## 3 Results

In the first round 23 delegates responded, 17 medical specialists, 3 professional patient representatives, 2 pharmacists and 1 nurse specialist, all with expertise in CPG development and knowledge of one or more rare diseases. All 6 proposed criteria were assessed as important by most of the respondents, and 64 additional criteria were proposed (see Supporting Information S1: Appendix 1).

In the second round, 19 panel members responded. Of the 70 criteria, 16 criteria ended up with a combination score ≥ 10. This number was reduced to 12 after the removal of duplicates. Of the six originally proposed criteria, all but one (approval by a relevant patient organization) had a combined score ≥ 10.

In the third round, 18 individual panel members responded, and 1 organization (Association of Innovative Medicines, VIG) returned responses from 5 individuals. The replies of this organization were combined by using the median of their ranks for each criterion, which led to 19 responses in total. The histograms showing the ranks for 12 criteria are shown in the Supporting Information S1: Appendix 1. The range of ranks varied from 6 for 1 criterion (‘A procedure for revising the guidance document has been stated’) to 11 for 1 criterion (‘Involvement of all relevant stakeholders’), while the maximum possible range for 12 criteria was 11.

Seven delegates attended the online consensus meeting and discussed the results of the third round. Several criteria were re‐formulated and combined until consensus was reached on seven final criteria.

Final criteria:

1. Goal and intended users are clearly described.
2. All relevant parties represented, including at least:
  (i) Patient representatives
  (ii) Expertise centre involved (if available)
  (iii) Involved healthcare professionals
3. Literature search and selection are transparent.
4. Taking inventory of problem areas as a basis for specific clinical questions.
5. The document has a number of recognizable components:
  (i) Authors and methods are described transparently
  (ii) Explicit description of methods for formulating recommendations
  (iii) The strengths and limitations of the body of evidence are clearly described
  (iv) Key recommendations are easily identifiable
6. Competing interests of guideline development group members have been recorded and addressed.
7. The guideline has been externally reviewed and authorized by predetermined parties prior to its publication.

## 4 Discussion

In this study, we have reached consensus with a broad group of stakeholders about a minimum set of criteria for the appraisal of guidance documents for rare diseases. Although beforehand we hoped that this minimum set of criteria might reduce the time and effort needed for the development of CPGs for rare diseases compared to common diseases, the need for methodological rigour perceived by the participants prevailed. Six of the seven criteria resulting from this Delphi procedure are consistent with the AGREE II criteria. The seventh criterion, ‘Taking inventory of problem areas as a basis for specific clinical questions’ denotes a step that is usually taken early in the guideline development process as it is performed by the Knowledge Institute. Involving all relevant stakeholders in this inventory enhances the implementation of the final recommendations. This step is essential for guideline development panels to focus on the questions with the highest priority in the clinical field, thus making optimal use of limited resources. Although this criterion does not concern methodological issues or reporting of CPGs, and it is not included in AGREE II, awareness of limitations in time and funding is increasingly important, albeit not exclusively in rare diseases.

In this Delphi procedure, the goal was to create a minimum set of criteria. However, during the process, the participants perceived it difficult to limit the number of methodological criteria for a CPG assessment. Throughout the Delphi process, careful consideration was given to the alignment of the new set of minimum criteria for a guidance document for rare diseases with the AGREE II criteria. During the first round, all AGREE II criteria were considered important to add to the prespecified set. The study by Hilton Boon et al. [15] also shows that AGREE II can be used for rare disease guidelines. However, the authors state that using AGREE II for rare disease guidelines also has challenges, such as the potential lack of a range of available treatment options or a scarcity of experts for the condition.

The AGREE II criteria were developed to evaluate the methodological rigour and transparency of the guideline development process [12]. It does not consider factors such as the decision‐making process determining when or for what condition a guideline should be developed, the sources of funding supporting its development, or the identification of specific problem areas to be addressed within the guideline. However, particularly when it comes to rare diseases, prioritization of topics is essential. Developing guidelines is resource‐intensive, particularly in terms of time investment, requiring careful consideration regarding which topics merit guideline development. This issue becomes more prominent in the context of rare diseases due to the large number of conditions, small patient populations, and limited existing knowledge [16]. Deciding for which rare diseases a CPG is warranted poses a unique challenge. The focus should be on rare diseases for which guidance documents provide significant value in reducing unwarranted practice variation. Therefore, prioritizing rare diseases that have the highest need for a guidance document is necessary. The new criterion of prioritizing problem areas was considered very important by the experts in the Delphi study. However, this is already standard procedure of the Knowledge Institute of the Dutch Association of Medical Specialists for developing CPGs, so for most panel members this is familiar. The issue of limited resources is outside the scope of the AGREE II criteria, but its relevance is not limited to rare diseases. When appraising a CPG, methodological rigour is important, but so is the relevance for patients and professionals of the topics addressed by the recommendations.

In this Delphi study, we have tried to actively involve all relevant stakeholders, such as patient organizations, paediatricians, clinical geneticists, gynaecologists, internal medicine specialists, neurologists, and the guidelines advisory committee of the Dutch Association of Medical Specialists. The active involvement of all relevant stakeholders, including patient representatives, contributes to the credibility and relevance of the criteria identified in this study. This patient‐centred approach aligns with the core principles of healthcare, emphasizing the importance of including the voices and experiences of patients in guidance document development [17]. Particularly in the development steps of prioritization of guidance documents for rare diseases, the active involvement of patient representatives is key. This ensures that the guideline addresses issues that are relevant to the field.

Contextual limitations of this Delphi study conducted within the Netherlands need to be acknowledged. Generalizing the findings to other regions or populations may be restricted. Replicating this Delphi study with a diverse and international range of stakeholders would provide valuable insights and enhance the robustness of the criteria for guidance documents for rare diseases. Modifying established tools by adding new items may influence their measurement properties. One might say that the ‘new’ set of criteria should be subjected to research into its measurement properties. However, it is not the intention to change the AGREE II tool for assessing the methodological quality and transparency of CPGs, since the criterion proposed by the Delphi panel is aimed at what questions to address instead of the way the recommendations should be developed.

In conclusion, the criteria developed through this Delphi study provide a foundation for the development of high‐quality guidance documents for rare diseases. These criteria may serve as a valuable resource and guide for developers, ensuring that future guidance documents for rare diseases are evidence‐based, comprehensive, and focused on the most relevant clinical questions.

## Author Contributions

**Iméze Jorieke Hieltjes:** writing – original draft, investigation, conceptualization. **Charlotte M. W. Gaasterland** and **Johanna H. van der Lee:** conceptualization, investigation, writing – review and editing. **Roel Bakx, Silvia C. W. van Breukelen, Robbert J. H. Ensink, Charlotte van Esch, Raoul C. M. Hennekam, Ilse van Herk, Marieke Hermsen, Anja M. C. Horemans, Hester Rippen, Bart P. C. van de Warrenburg** and **Anne‐Marie van Wermeskerken:** investigation.

## Conflicts of Interest

The authors declare no conflicts of interest.

## Supporting information

Appendix 1 supplements Delphi rounds. Appendix 2 histograms of ranks per criterium 2

An additional criterion to AGREE II to increase the efficiency of guideline development for rare diseases based on the results of a national Delphi study

## Data Availability

The data that support the findings of this study are available in the Supporting Material of this article.
