# Supplementary material for: An Additional Criterion to Appraisal of Guidelines for Research and Evaluation II Might Increase the Efficiency in Guideline Development for Rare Diseases: Results of a National Delphi Study
Source: Clin Public Health Guidel. 2025 Sep 7;2(4):e70040. doi: 10.1002/gin2.70040 (PMC13563410; doi:10.1002/gin2.70040)
Supplement: Supplementary file 1 [file GIN2-2-e70040-s001.docx]

**An additional criterion to AGREE II might increase the efficiency in guideline development for rare diseases; results of a national Delphi study – Appendix 1**

Histograms of ranks per criterion (low rank means high priority); third round

Mean = 3,79

Median = 2

Std. Dev. = 3,119

N = 19

Mean = 4,37

Median = 4

Std. Dev. = 2,385

N = 19

Mean = 5

Median = 4

Std. Dev. = 2,789

N = 19

Mean = 5,11

Median = 5

Std. Dev. = 2,492

N = 19

Mean = 5,11

Median = 4

Std. Dev. = 3,526

N = 19

Mean = 6

Median = 6

Std. Dev. = 2,848

N = 19

Mean = 6,21

Median = 6

Std. Dev. = 3,691

N = 19

Mean = 7,16

Median = 6

Std. Dev. = 2,853

N = 19

Mean = 7,89

Median = 8

Std. Dev. = 3,053

N = 19

Mean = 8,21

Median = 9

Std. Dev. = 3,409

N = 19

Mean = 8,47

Median = 9

Std. Dev. = 2,342

N = 19

Mean = 10,58

Median = 11

Std. Dev. = 1,575

N = 19
