# Supplementary material for: An Additional Criterion to Appraisal of Guidelines for Research and Evaluation II Might Increase the Efficiency in Guideline Development for Rare Diseases: Results of a National Delphi Study
Source: Clin Public Health Guidel. 2025 Sep 7;2(4):e70040. doi: 10.1002/gin2.70040 (PMC13563410; doi:10.1002/gin2.70040)
Supplement: Supplementary file 2 [file GIN2-2-e70040-s002.docx]

An additional criterion to AGREE II to increase the efficiency of guideline development for rare diseases based on the results of a national Delphi study

Supplements

1.1. Supplement 1: Results from Round 1

| **Criteria (number of the AGREE II criterium with the most overlap)** | **Necessary for rare diseases?** | | **The importance of each criterium? (1= unnecessary; 10= essential)** |
| --- | --- | --- | --- |
|  | Round 1 number of ‘yes’ (from 23 respondents) | Round 1 comments | **Round 2 for grading** |
| 1. Authors and methods transparent (if no literature is demonstrably available, it is indicated that the document has been drawn up on a consensus basis)(10 from AGREE II) | 23 |  |  |
| 2. Involvement of all relevant parties (4 from AGREE II) | 22 | 1. I said no here because there are always primary and secondary authorizing parties. The involvement of a secondary party is not strictly necessary in terms of involvement, but we do consider it in the Authorisation process.  2. To explicitly mention patient representation here and consider health insurers. |  |
| 3. Code for the Prevention of Influence by Conflict of Interest Followed (23 from AGREE II) | 21 | 1. Although I don't think this should be a reason for exclusion from the working group in this case. Due to the small number of experts for rare conditions, the likelihood of some form of conflict of interest is high.  2. Complete absence of conflict of interest is not feasible nor desirable given the expertise and limited number of experts (in healthcare/research). However, a code of conduct can be signed to promote awareness. |  |
| 4. Systematically searched and selected literature (for efficiency, for example an overarching search per document, not per module, especially for disorders about which little is published, with an annual search update if necessary) (7 from AGREE II) | 23 | - I find the part between (_) not very clearly formulated and I don't fully understand it. - even desirable - there can still be some discussion about where to use an overarching search and where to use targeted searches - Yes, but in a more concise form - I really think the search is crucial, even if the expert group knows there is no literature, it is still good to do a search. - Provided that you already know in advance that there is no literature. Otherwise, by doing a search, you demonstrate that there is no literature. This contributes to transparency and support. |  |
| 5. Authorisation by relevant scientific associations (13 from AGREE II) | 22 | 1. National or international 2. For consensus and equal outcomes 3. Yes (majority); consideration for no: it concerns assessment by referees who are not involved in the working group, it is important to assess to what extent this is feasible if most experts and relevant PAGs in the field of rare diseases will already be working group members. |  |
| 6. Agreement of the Patient Federation or another relevant patient organisation (5 from AGREE II) | 19 | 1. Instead of 6: Consultation with relevant patient federation or other relevant patient organisations 2. (relevant patient organisations/difficult to be complete) 3. I find AGREEMENT too strong; in the AGREE II appendix it also states that 'The perspectives and preferences of the target population (patients/general population) have been sought.' That is something different. I think input/feedback will certainly be sought, but there may not be an association for every rare disease and an agreement from the Patient Federation would then feel artificial. 4. if available 5. This criterion does not correspond to criterion 5 of AGREE II. Criterion 5 of AGREE II is about 'the perspective' sought from the target population, not an 'agreement.' 6. Or a few patients if there is no organisation. Possibly in combination with the PF. |  |
| **Criteria that are added in round 1 (random order):** | | |  |
| 7. Publication in a peer-reviewed journal (that is not the same as stated at point 5 above). | | |  |
| 8. The perspective and preferences of the target population with regard to the topics to be discussed, plus the feasibility of research and treatment actions in the document, are strongly considered (that is not what is stated at point 6) | | |  |
| 9. The population to which the document applies is clearly defined | | |  |
| 10. It is clear for each recommendation how it is supported / supported by the authors of the document; drafters independently have the opportunity to express their opinion on each recommendation | | |  |
| 11. If the document is about a condition that can occur under different circumstances/severity/affected persons, the recommendations are formulated in such a way that they apply to all situations etc, or else it is specified when they are and when they are not applicable | | |  |
| 12. The purpose of the guideline is specifically described: agree II item 1 | | |  |
| 13. The intended users have been named: agree II item 6 | | |  |
| 14. More important than item 4 seems to me to be item 10: properly describing the method used to arrive at a recommendation | | |  |
| 15. The population (patients/general population) to which the guideline applies is specifically described (3 from AGREE II) | | |  |
| 16. The development of guidelines/standards starts with a bottleneck analysis. | | |  |
| 17. Specific basic questions are formulated on the basis of a bottleneck analysis. | | |  |
| 18. The method used to formulate the recommendations is explicitly described. | | |  |
| 19. The recommendations are specifically formulated | | |  |
| 20. The guideline/standard has a number of recognizable parts and a modular structure | | |  |
| 21. The guideline/standard is approved by at least all primary professional groups and patient/client organisation(s). | | |  |
| 22. The organisations primarily involved are committed to promoting the application of the guideline/standard in practice | | |  |
| 23. (3) The population (patients/general population) to which the guideline applies is specifically described | | |  |
| 24. (6) The intended users of the Directive are clearly identified. | | |  |
| 25. (8) The criteria for selecting the scientific evidence are clearly described. | | |  |
| 26. (9) The strengths and limitations of the scientific evidence are clearly described. | | |  |
| 27. (13) The guideline has been reviewed by external experts before publication. -> There has been a round of comments among relevant professional groups, patient groups and external parties. | | |  |
| 28. (14) A procedure for revision of the Directive is mentioned. | | |  |
| 29. (16) The different policy options are clearly stated. | | |  |
| 30. (17) The core recommendations are easy to recognize. | | |  |
| 31. (20) The possible implications of applying the recommendations for costs and resources have been considered. | | |  |
| 32. (Domain 3. 11) Health gain, outcome measures of eg survival and quality of life. | | |  |
| 33. (Domain 4.15) Recommendations: specific and unambiguous. Recognizable intent or purpose. E.g. Quality of life. | | |  |
| 34. (Domain 2.4) The members of the working group that develop the guidelines come from all relevant professional groups. So relevant expertise. | | |  |
| 35. I think the question and purpose of the document should be clearly defined as far as I am concerned, especially in the case of rare disorders | | |  |
| 36. (3) The population (patients/general population) to which the guideline applies is specifically described. | | |  |
| 37. (12) There is an explicit link between the recommendations and the underlying evidence. | | |  |
| 38. For many themes, recommendations will result in expert opinion, so a criterion for this should be added. | | |  |
| 39. Purpose of the document is specifically described (health goals (diagnosis and/or treatment, etc.), (1 from AGREE II) This is due to findability. | | |  |
| 40. The question(s) addressed is/are specifically described; Target group, intervention(s) or exposure, comparative interventions or factor(s), if applicable, outcome(s) setting in health care or context. (2 from AGREE II) | | |  |
| 41. The strengths and limitations of the scientific evidence are described. (9 from AGREE II). I classify the application of GRADE here. | | |  |
| 42. A statement on the revision of the directive (14 from AGREE II) This is related to the intended annual update | | |  |
| 43. (possibly. The guideline describes how the guideline working group has assessed the scientific evidence and linked it to the recommendations.. (12 from AGREE II) EtD argumentation and weighting. This seems to me a necessary condition for the annual update, but may also be ranked under 10 from AGREEII (criterion 1 above)) | | |  |
| 44. (19) The Directive provides advice and/or tools for applying the recommendations in practice. | | |  |
| 45. For the pharmaceutical module (therapy): only include substances registered in the Netherlands? Also include reimbursement status? (wherever you can refer to www.kinderformularium.nl as a source to be consulted for the dosage to be used) | | |  |
| 46. (11) Health benefits, side effects and risks have been considered when preparing the recommendations. | | |  |
| 47. (17) The core recommendations are easy to recognize. | | |  |
| 48. (2) The question(s) addressed in the Directive is/are specifically described. | | |  |
| 49. (3) The population (patients/general population) to which the guideline applies is specifically described. | | |  |
| 50. A procedure for revision of the directive is mentioned (14 from AGREE II) | | |  |
| 51. Health gains, side effects and risks were considered when drawing up the recommendations (11 from AGREE II) | | |  |
| 52. The intended users of the guideline are clearly identified (6 from AGREE II) and work in a recognized expertise center for the disease to which the guideline applies. | | |  |
| 53. The organisation of care for the clinical picture as described in the guideline has a single point of contact in the treatment expertise center | | |  |
| 54. The purpose of the guideline (now the quality document) is specifically described (with the different policy options). | | |  |
| 55. The guideline (now the quality document) describes the facilitating and hindering factors in its application. | | |  |
| 56. Authorisation by relevant scientific associations and/or relevant expertise centers recognized by the Ministry of Health, Welfare and Sport | | |  |
| 57. Suitable for further elaboration in care pathways, which are conditional for the recognition by VWS of expertise centers | | |  |
| 58. Agreement of relevant European Reference Network (ERNs) | | |  |
| 59. Document deals with integrated care. | | |  |
| 60. formulate outcome measures and instruments only by consensus | | |  |
| 61. Formulating bottlenecks | | |  |
| 62. shaping national/international collaboration in care and research substantively for the workplace | | |  |
| 63. joint agreement/consensus about care/research protocol | | |  |
| 64. of course also everything from domain 1. | | |  |
| 65. The criteria for selecting the scientific evidence are clearly described. | | |  |
| 66. The strengths and limitations of the scientific evidence are described. May also be; there is only observational data. | | |  |
| 67. The potential implications of applying the recommendations for costs and resources have been considered. | | |  |
| 68. The views of the funding body have not influenced the content of the Directive. | | |  |
| 69. Conflicting interests of guideline working group members have been identified and discussed | | |  |
| 70. The purpose of the quality document is specifically described | | |  |

1.2. Supplement 2: Criteria from round 3 with prioritization from the participants

1. The goal of the quality document is specifically described.
2. Systematically searched and selected literature (for efficiency, for example, an overarching search per document, not per module, especially for diseases with little published literature, with an annual search update if necessary).
3. Specific starting questions are formulated based on a bottleneck analysis.
4. Authors and methods are transparent (when there is demonstrably no literature available, it is indicated that the document is based on consensus) / The process by which the recommendations are developed is explicitly described.
5. Involvement of all relevant parties.
6. The strengths and limitations of the scientific evidence are clearly described.
7. The core recommendations are easily recognizable.
8. The intended users are named.
9. A code to prevent undue influence from conflicts of interest is followed.
10. The document has a number of recognizable sections and a modular structure.
11. The document has been reviewed for publication by external experts.
12. A procedure for revising the quality document is mentioned.
